# Sex differences in the association between spousal metabolic risk factors with incidence of type 2 diabetes: a longitudinal study of the Iranian population

**DOI:** 10.1186/s13293-019-0255-5

**Published:** 2019-08-22

**Authors:** Azra Ramezankhani, Kamran Guity, Fereidoun Azizi, Farzad Hadaegh

**Affiliations:** 1 Prevention of Metabolic Disorders Research Center, Research Institute for Endocrine Sciences, Shahid Beheshti University of Medical Sciences, Tehran, Iran; 2 Cellular and Molecular Endocrine Research Center, Research Institute for Endocrine Sciences, Shahid Beheshti University of Medical Sciences, Tehran, Iran; 3 Endocrine Research Center, Research Institute for Endocrine Sciences, Shahid Beheshti University of Medical Sciences, Tehran, Iran

**Keywords:** Metabolic risk factors, Spouse, Type 2 diabetes

## Abstract

**Background:**

We investigated whether metabolic risk factors in one spouse were associated with an excessive risk of type 2 diabetes in the other.

**Methods:**

The study cohort (1999–2018) included 1833 men and 1952 women, aged ≥ 20 years with information on both their own and their spouse’s diabetes status and metabolic risk factors including body mass index (BMI), waist circumference, systolic and diastolic blood pressure, triglyceride to high-density lipoprotein cholesterol ratio, and type 2 diabetes. The associations between spousal metabolic risk factors and type 2 diabetes were estimated using Cox regression models adjusted for the three nested sets of covariates.

**Results:**

We found 714 (360 men and 354 women) incident cases of type 2 diabetes, after more than 15 years of follow-up. Among women, having a husband with diabetes was associated with a 38% (hazard ratio (HR) 1.38; 95% confidence interval (CI) 1.03, 1. 84) increased risk of type 2 diabetes, adjusted for age, socioeconomic status, individual’s own value of the respective spousal exposure variable, family history of diabetes, and physical activity level. After further adjustment for the woman’s own BMI level, the husband’s diabetes was associated with 23% (HR 1.23; 0.92, 1.64) higher risk of type 2 diabetes in wives, values which did not reach statistical significance. No significant associations were found between spousal metabolic risk factors and incidence of type 2 diabetes among index men.

**Conclusion:**

We found a sex-specific effect of spousal diabetes on the risk of type 2 diabetes. Having a husband with diabetes increased an individual’s risk of type 2 diabetes. Our results might contribute to the early detection of individuals at high risk of developing type 2 diabetes, particularly, in women adversely affected by their partner’s diabetes.

## Background

Diabetes is a common chronic disease that is rapidly increasing worldwide. It is projected that the number of people with diabetes worldwide will rise by 592 million by 2035. More than 80% of people with diabetes live in low- and middle-income countries [1]. In Iran, the annual incidence of type 2 diabetes is estimated to be 1%, accounting for more than 800,000 new cases each year [2]. Diabetes is a multi-factorial disorder that develops as a result of complex interactions between multiple genes and environmental/behavioral factors [3, 4]. Genome-wide association studies have identified 144 genetic variants at 129 loci affecting type 2 diabetes risk, although the contribution of these variants to the heritability of type 2 diabetes is generally small (~ 10%) [5]. On the other hand, a number of environmental and lifestyle factors such as an energy-dense diet, low physical activity, a sedentary lifestyle, emotional stress, and socioeconomic status have been suggested to be highly influential and are also critical for the expression of genetic risk [4, 6]. A growing number of studies have shown association between couples in a range of health problems such as hypertension [7], coronary heart disease [8], and mental, physical, and social health outcomes [9]. Couples share the same environment which may increase risk of disease concordance [10]. A concordance in disease risk has also been shown for diabetes [6, 11, 12]. The pooled analysis of 17 studies, including 3 case-control, 5 cohort, and 9 cross sectional studies suggested a positive association between history of diabetes in one spouse and risk of type 2 diabetes in the other spouse [11]. A recent study of adults has shown that a wife’s obesity increased the risk of type 2 diabetes among men, but not vice versa [12]. Hence, the presence of metabolic risk factors in a spouse might be used as an important predictor of individual risk for type 2 diabetes. Current cohort studies on the spousal correlations for type 2 diabetes have been carried out in the USA, UK, and Sweden [11–15]. However, since the impact of socio-environmental factors may differ between countries, their findings may not be generalizable to other populations. Therefore, in a longitudinal study, we investigated how the risk of type 2 diabetes would be affected in Iranian couples in which one spouse had type 2 diabetes and the other metabolic risk factors.

## Methods

### Study population

The Tehran lipid and glucose study (TLGS) is a large population-based cohort study initially designed to investigate the risk factors and outcomes for non-communicable disease. The TLGS study design has previously been published [16]. In brief, during 1999–2002 (first phase) and 2002–2005 (second phase), a total of 15,005 and 3551 people aged ≥ 3 years, respectively, were enrolled and were followed in the next phases (phase 2, 2002–2005; phase 3, 2005–2008; phase 4, 2008–2011; phase 5, 2012–2015; and phase 6, 2015–2018). To eliminate the bias of self-reported measures of marital status, identification of couples in this study was performed using a genealogy database. In the TLGS, genealogy data were collected based on households and we assumed that the unit of the family included parents and at least one child; therefore, we added “dummy persons” to replace missing parents. For this study, we selected 12,790 participants aged ≥ 20 years (10,362 individuals from the first phase and 2428 participants from the second phase). We excluded the couples who did not have any child after their marriage, those that had gotten married before the age of 18 years, couples who did not participate in any phases of the TLGS, and those who did not participate simultaneously at the same phase, leaving us 2866 couples. All analyses were performed separately for men and women (index individuals); hence, of 2866 couples, we excluded “index individuals” with prevalent diabetes at baseline (350 men and 296 women), those with missing data on diabetes status at baseline (151 men and 74 women), missing data on other covariates (161 men and 235 women), and those with no follow-up data after recruitment until the end of the study (18 April 2018) (370 men and 309 women). Consequently, a total of 3785 index individuals (1833 men and 1952 women) remained in the study. Family structures and drown pedigrees were confirmed by genomic data from the Tehran Cardiometabolic Genetic Study (TCGS) [17]. This study was approved by the ethics committee of the Research Institute for Endocrine Sciences of Shahid Beheshti University of Medical Sciences, Tehran, Iran, and was conducted according to the principles of the Declaration of Helsinki. All participants provided written consent prior to any study procedures.

### Data collection

Participants completed a baseline questionnaire including demographic and clinical characteristics such as age, sex, marital status, education level, medication use, smoking, prevalent cardiovascular disease (CVD), and family history of diabetes mellitus (FH-DM). Waist circumference (WC), weight, and height were measured based on the standard protocols [16], and body mass index (BMI) was calculated as weight (kilogram) divided by square of height (square meters). Systolic (SBP) and diastolic blood pressure (DBP) were obtained by the mean of two measurements taken on the right arm at an interval of 5 min. The participants’ blood samples were collected after an overnight fast of 12–14 h to assess the fasting plasma glucose (FPG), 2 h post load plasma glucose (2 h-PLPG), triglyceride (TG) and high-density cholesterol (HDL-C) level [16]. Triglyceride to high-density lipoprotein cholesterol ratio (TG/HDL-C) was calculated by dividing TG to HDL-C. Physical activity level (PAL) was assessed using the Lipid Research Clinic questionnaire in the first phase of the study. In the second phase, the Modifiable Activity Questionnaire was used to measure three forms of activities including leisure time, job, and household activities in the past year [18].

### Definition of terms

Education level was categorized into 3 levels: < 6 years, 6–12 years, and > 12 years of schooling. The highest level of educational attainment, at the couple level, was defined as a proxy for socioeconomic status (SES) and was used for both spouses. A current smoker was defined as a person who was smoking cigarettes daily or occasionally or those who used other tobacco products. A past-smoker was a person who had smoked daily or occasionally and those who had quit smoking. Never smokers were defined as people who had never smoked before. Family history of diabetes (FH-DM) was defined as having type 2 diabetes in first-degree relatives. Prevalent CVD was defined as a self-reported history of specific types of CVD including coronary heart disease (CHD) or stroke. Low physical activity was defined as doing exercise or labor less than three times a week or scores ≤ 600 MET (metabolic equivalent task)-minutes per week [19].

### Exposures

The main exposures in our study were spouse’s metabolic risk factors at baseline including type 2 diabetes, BMI, WC, SBP and DBP, and TG/HDL-C. All exposures were considered as continuous measures, except type 2 diabetes.

### Outcome

The examined outcomes included the first occurrence of type 2 diabetes during the follow-up period. Type 2 diabetes was defined as FPG ≥ 7 mmol/L or 2 h-PLPG ≥ 11.1 mmol/L [20] or using glucose-lowering treatment.

### Statistical methods

Comparison of baseline characteristics of the index individuals was done using Student’s *t* test and Chi-square test, as appropriate. Additionally, we assessed the statistical dependence between the study variables in the two spouses using the Pearson and Spearman correlation tests for continuous and categorical variables, respectively.

Comparison of baseline characteristics between respondents (index individuals with complete data at baseline who had at least one follow-up data) and non-respondents (those with missing data at baseline or without any follow-up data) was performed by Student’s *t* test for continuous variables and the Chi-square test for categorical variables.

Incidence density and 95% confidence interval (CI) for type 2 diabetes were calculated per 1000 person-years. Cox proportional hazard regression was used to calculate the association between spouse’s metabolic factors and incident type 2 diabetes. The event date was defined as the mid-time between the date of the follow-up visit when the diagnosis of diabetes was made for the first time and the most recent follow-up visit prior to the diagnosis. Survival time was computed as the time between baseline and the event date (for incident cases) or the last follow-up (for censored cases). Individuals were censored due to death from a cause other than diabetes, loss to follow-up, or the end of the study (18 April 2018) without the event occurring.

Four models were developed: Model 1 was adjusted for age, model 2 was further adjusted for SES, and in model 3, we further adjusted for the index individual’s own value of the respective spousal exposure variable, FH-DM and PAL. Models including blood pressure (SBP and DBP) or lipid levels (TG/HDL-C) were further adjusted for antihypertensive and lipid-lowering medications, respectively. Finally, in model 4, we further adjusted for each index individual’s own value of BMI. Models including type 2 diabetes as exposure were adjusted for the value of index individual’s FPG.

Proportional hazards assumptions in the Cox models were checked using statistical tests based on the scaled Schoenfeld residuals and log-log plots, indicating all proportionality assumptions were appropriate. All statistical analysis was performed using the R statistical package, v.3.4.0 (www.r-project.org); Two-tailed *p* values < 0.05 were considered statistically significant.

## Results

The study population consisted of 3785 index individuals (1833 men and 1952 women) with the mean (SD) ages of 45.8 (12.3) and 39.7 (11.1) years, respectively. Baseline characteristics of the index individuals are shown in Table 1. In general, index individuals in both sexes were predominantly of middle and high SES. Index men had higher levels of WC, SBP, DBP, FPG, and TG/HDL-C and were more likely to be smokers, compared with index women (all *p* values < 0.001). Moreover, among the original population (2866 couples), the mean (SD) number of children per family was 1.6 (1.2). Additionally, the mean (SD) age of children per family was 15.6 (8.6). The mean (SD) of the child sex ratio (the ratio of males to females) was 51.1 (39.9) per family.

**Table 1:** Baseline characteristics of index individuals stratified by sex; Tehran Lipid and Glucose study (TLGS) (1999–2018)

	Men *n* = 1833	Women *n* = 1952
Continuous variables		
Age (years)	45.8 (12.3)	39.7 (11.1)
BMI (kg/m^2^)	26.1 (3.7)	27.6 (4.5)
Waist circumference (cm)	89.9 (10.3)	87.3 (11.6)
SBP (mmHg)	119.1 (17.1)	115.1 (16.9)
DBP (mmHg)	77.7 (10.8)	76.5 (10.3)
FPG (mmol/L)	5.07 (0.5)	4.9 (0.5)
2 h-PLPG (mmol/L)	5.8 (1.7)	6.1 (1.5)
TG (mmol/L)	2.1 (1.3)	1.7 (1.0)
HDL-C (mmol/L)	0.4 (0.1)	0.5 (0.1)
TG/HDL-C	5.4 (4.7)	3.6 (2.9)
Categorical variables		
Spousal diabetes	157 (8.5)	215 (11.0)
SES		
< 6 years of schooling	330 (18.0)	350 (17.9)
6–12 years of schooling	1088 (59.3)	1175 (60.1)
> 12 years of schooling	415 (22.6)	427 (21.8)
Family history of diabetes	457 (24.9)	538 (27.6)
Prevalent CVD	90 (4.9)	50 (2.6)
Smoking status		
Never	1005 (54.8)	1853 (94.9)
Past	270 (14.7)	31 (1.6)
Current	558 (30.4)	68 (3.5)
Physical activity level		
Low physically active	450 (24.5)	529 (27.1)
Lipid-lowering medication use	35 (1.9)	45 (2.3)
Antihypertensive medication use	72 (3.9)	107 (5.5)

Values are presented as mean (standard deviation) for continuous variables and, frequency (percent) for categorical variables

*BMI* body mass index; *CVD* cardiovascular disease; *FPG* fasting plasma glucose, *SBP* systolic blood pressure; *DBP* diastolic blood pressure; *TG/HDL-C* triglyceride to high-density lipoprotein cholesterol ratio; *2 h-PLPG* 2 h post load plasma glucose; *SES* socioeconomic status defined as the highest level of educational attainment, at the couple level; *SD* standard deviation

Comparisons between non-respondents and respondents are shown in Table 2. Generally, there were no significant differences in the baseline characteristics of respondent and non-respondent among women, except for BMI. Female respondents had a lower baseline BMI, compared with their non-respondent counterparts. Among men, respondents had a higher probability of being a current smoker (*p* < 0.001), but a lower probability of experiencing a CVD event (*p* < 0.01), compared to their non-respondent counterparts.

**Table 2:** Baseline characteristics of respondents and non-respondents stratified by sex; Tehran Lipid and Glucose study (TLGS) (1999–2018)

	Women			Men		
Variables	Respondent *n* = 1952	Non-respondent *n* = 618	*P* value	Respondent *n* = 1833	Non-respondent *n* = 683	*P* value
Age (years)	39.7 (11.1)	38.9 (11.6)	0.173	45.8 (12.3)	46.5 (13.4)	0.248
BMI (kg/m2)	27.6 (4.5)	28.2 (5.1)	0.022	26.1 (3.7)	25.9 (4.1)	0.250
Waist circumference (cm)	87.3 (11.6)	88.4 (12.3)	0.066	89.9 (10.3)	89.1 (10.9)	0.092
SBP (mmHg)	115.1 (16.9)	115.3 (18.8)	0.851	119.1 (17.1)	119.9 (18.8)	0.354
DBP (mmHg)	76.5 (10.3)	75.8 (11.2)	0.183	77.7 (10.8)	78.1 (11.4)	0.575
TG/HDL-C	3.6 (2.9)	3.9 (4.2)	0.085	5.4 (4.7)	5.2 (4.2)	0.411
FPG (mmol/L)	4.9 (0.5)	4.9 (0.5)	0.940	5.1 (0.5)	5.1 (0.5)	0.975
Education						
< 6 years of schooling	549 (28.1)	178 (28.8)	0.878	452 (24.6)	185 (27.1)	0.457
6–12 years of schooling	1225 (62.8)	381 (61.7)		1044 (57.0)	375 (54.9)	
> 12 years of schooling	178 (9.1)	59 (9.5)		337 (18.4)	123 (18.0)	
Family history of diabetes	538 (27.6)	170 (27.5)	0.975	457 (24.9)	164 (24.0)	0.634
Prevalent CVD	50 (2.6)	17 (2.8)	0.773	90 (4.9)	52 (7.6)	0.011
Smoking status						
Never	1854 (95.0)	577 (93.5)	0.361	864 (47.1)	374 (54.8)	< 0.001
Past	30 (1.5)	14 (2.2)		240 (13.1)	101 (14.7)	
Current	68 (3.5)	27 (4.3)		729 (39.8)	208 (30.5)	
Physical activity level						
Low physically active	1421 (72.8)	472 (76.4)	0.090	1338 (72.9)	464 (67.9)	0.666
Lipid-lowering medication	30 (1.5)	13 (2.1)	0.368	20 (1.1)	8 (1.2)	0.833
Antihypertensive medication	85 (4.4)	31 (5.0)	0.505	64 (3.5)	35 (5.1)	0.061

Mean (SD) are shown for continuous variables and *P* value is calculated with *t* test; frequency (%) are shown for categorical variables with *P* value based on chi-square test

*BMI* body mass index, *CVD* cardiovascular disease, *FPG* fasting plasma glucose, *SBP* systolic blood pressure, *DBP* diastolic blood pressure, *TG/HDL-C* triglyceride to high-density lipoprotein cholesterol ratio, *SD* standard deviation

Pearson’s correlation coefficients between the same risk factors in the couples were BMI 0.12, WC 0.24, SBP 0.31, DBP 0.15, HDL-C 0.10, FPG 0.13, 2 h-PLPG 0.15 (*p* < 0.001 for all), and TG 0.06 (*p* < 0.01). Spearman’s correlation coefficients were antihypertensive medication use 0.09, lipid-lowering medication use 0.06, prevalent CVD 0.08, smoking status 0.09 (*p* < 0.001 for all), and PAL 0.04 (*p* < 0.05).

The median (interquartile range) of follow-ups was 15.4 (12.1–16.6) years and 15.5 (12.8–16.5) years in index men and women, respectively. We found 714 (360 men and 354 women) incident cases of type 2 diabetes during follow-up. The incidence rate (95% CI) per 1000 person-years were 15.4 (13.8–17.1) and 13.7 (12.3–15.2) in index men and women, respectively.

Table 3 displays the estimated relationships between spousal risk factor and incidence of type 2 diabetes for men and women. Among index men, after adjusting for age and SES, we found an association between the levels of spouse’s BMI (hazard ratio (HR) 1.02; 95% confidence interval (CI) 1.00, 1.05), SBP (1.01; 1.00, 1.01) and DBP (1.01; 1.00, 1.02) and the risk of type 2 diabetes (model 2). However, no significant risk remained after further adjustment for the men’s risk factor and additional confounders (models 3 and 4). Among women, the risk of type 2 diabetes increased with their husband’s WC (1.01; 1.00, 1.01) in the age and SES-adjusted model (model 2), and the association disappeared after further adjustment for the women’s risk factor and additional confounders (models 3 and 4). Furthermore, we observed 38% increased risk of type 2 diabetes (1.38; 1.03–1.84) in women whose husbands had type 2 diabetes, adjusted for the woman’s own FPG level, FH-DM, and PAL. After further adjustment for a woman’s own BMI level (model 4), the husband’s diabetes was associated with a 23% higher risk of type 2 diabetes in wives, values which did not reach statistical significance.

**Table 3:** The influence of spousal risk factors on type 2 diabetes development; Tehran Lipid and Glucose study (TLGS) (1999–2018)

Spousal risk factor	Model 1	Model 2	Model 3	Model 4
Men (*n* = 1833)				
BMI (kg/m^2^)	1.02 (1.00–1.05)*	1.02 (1.00–1.05)*	1.01 (0.99–1.03)	–
Waist circumference (cm)	1.01 (0.99–1.01)	1.01 (0.99–1.01)	1.00 (0.99–1.01)	1.00 (0.99–1.01)
SBP (mmHg)	1.00 (0.99–1.01)*	1.01 (1.00–1.01)*	1.00 (0.99–1.01)	1.00 (0.99–1.01)
DBP (mmHg)	1.01 (1.00–1.02)*	1.01 (1.00–1.02)*	1.00 (0.99–1.01)	1.00 (0.99–1.01)
TG/HDL-C	1.05 (0.98–1.13)	1.05 (0.98–1.13)	1.03 (0.96–1.11)	1.02 (0.95–1.10)
Type 2 diabetes	1.07 (0.75–1.53)	1.06 (0.74–1.52)	1.03 (0.72–1.48)	1.05 (0.73–1.50)
Women (*n* = 1952**)**				
BMI (kg/m^2^)	1.02 (0.99–1.05)	1.02 (0.99–1.05)	1.00 (0.97–1.03)	–
Waist circumference (cm)	1.01 (1.00–1.02)*	1.01 (1.00–1.02)*	1.00 (0.99–1.01)	1.00 (0.99–1.01)
SBP (mmHg)	0.99 (0.98–1.00)	0.99 (0.98–1.00)	0.99 (0.98–1.00)	0.99 (0.99–1.00)
DBP (mmHg)	0.99 (0.99–1.00)	0.99 (0.99–1.00)	0.99 (0.98–1.00)	0.99 (0.99–1.00)
TG/HDL-C	1.01 (0.97–1.04)	1.01 (0.97–1.04)	0.99 (0.96–1.04)	0.99 (0.95–1.03)
Type 2 diabetes	1.34 (1.01–1.79)*	1.33 (1.00–1.78)*	1.38 (1.03–1.84)*	1.23 (0.92–1.64)

Model 1**,** adjusted for age

Model 2**,** adjusted for factors in model 1 + SES

Model 3**,** adjusted for factors in model 2 + index individual’s risk factor + FH-DM + PAL. Models including blood pressure (SBP and DBP) or lipid levels (TG/HDL-C) were further adjusted for antihypertensive and lipid-lowering medication, respectively

Model 4**,** adjusted for factors in model 3 + index individual’s BMI

Models including type 2 diabetes, as exposure, were adjusted for the value of index individual’s FPG

*Significant results at the level of 0.05

*BMI* body mass index, *FH-DM* family history of diabetes mellitus, *PAL* physical activity level, *FPG* fasting plasma glucose, *SBP* systolic blood pressure, *DBP* diastolic blood pressure, *TG/HDL-C* triglyceride to high-density lipoprotein cholesterol ratio, *SES* socioeconomic status defined as the highest level of educational attainment, at the couple level

## Discussion

In this community-based study, we showed that spousal BMI, SBP, and DBP were associated with the risk of type 2 diabetes among men, adjusted for age and SES. These relationships disappeared when FH-DM, PAL, and the man’s own risk factors were adjusted for. Among women, we observed 38% increased risk of type 2 diabetes in women whose husbands had type 2 diabetes, after adjustment for age, SES, FH-DM, PAL, and the woman’s own FPG level. Interestingly, after further adjustment for the woman’s own BMI, the husband’s diabetes was associated with a 23% higher risk of type 2 diabetes in wives, which did not reach statistical significance.

In a recently published study, Nielsen et al. [12] investigated associations between spousal metabolic risk factors and incidence of type 2 diabetes among 3649 men and 3478 women. They showed that each 1-kg/m^2^ increase in wives’ BMI was associated with a 4% increased risk of type 2 diabetes in the husbands, even after adjustment for the man’s own BMI level. Also, they found a positive relation between triacylglycerol levels in husbands with a risk of type 2 diabetes in wives. In our study, the positive relation between the wives’ BMI and the risk of type 2 diabetes in the husbands disappeared after further adjustment for the husbands’ own BMI.

Associations between spousal diabetes and risk of diabetes have been investigated in some studies [11–15]; nevertheless, it is difficult to conduct a direct comparison of our results with theirs due to the different types of study designs, different strategies for inclusion of the study populations, and in particular, the different adjustments considered in their multivariate analysis. In a prospective study, Nielsen et al. [12] did not find any association between spousal diabetes with an increased risk of type 2 diabetes in the index individuals. Also, a study from the Framingham Offspring Study did not find a clearly increased risk of type 2 diabetes in spouses of diabetic individuals [13].

In this study, we found a higher risk of developing type 2 diabetes in women whose husbands had type 2 diabetes, even after adjustment for the woman’s own FPG level, an association that was attenuated after adjustment for the woman’s own BMI. This finding is supported by several studies; a large prospective study of 35 million Swedish families [14] found a 32% higher risk of developing type 2 diabetes in individuals with a spouse with type 2 diabetes; this value remained higher after adjustment for BMI. Furthermore, the recent study by Appiah [11] reported that adults who had a spouse with diabetes had a 20% increased risk for incident type 2 diabetes, compared to those whose spouse did not have the disease. A meta-analysis by Appiah et al. [11] summarizing the results of 17 published studies with prospective or cross sectional designs, showed that having a spouse with diabetes was significantly associated with developing diabetes (pooled OR 1.88, CI 1.52–2.33); they found that the pooled estimate did not vary after adjustment for BMI, diabetes diagnostic criteria, and study quality.

Two mechanisms have been suggested for explaining the spousal concordance of diabetes: (1) assortative mating, which refers to the fact that people typically choose a partner with similar characteristics, religion, socioeconomic positions, and lifestyle patterns, such as diet, physical activity, smoking, and alcohol consumption, and (2) shared lifestyle patterns, suggesting that concordance may be due to shared environmental risk factors, resources, social habits, eating patterns, PALs, and other health behaviors, all of which contribute to convergence on such key health behaviors [6, 21, 22]. In our study, spousal diabetes as a risk factor for the development of type 2 diabetes among the women cannot be fully explained by the assortative mating theory because, the observed association did not drastically diminish when we further adjusted for woman’s own BMI (as a proxy for assortative mating and part of the causal pathway); this finding suggests that the observed association might be mediated by other unhealthy behaviors, such as physical inactivity and unhealthy eating habits which are the strong risk factors for type 2 diabetes [23, 24].

Our study suggests the predictive role of spousal diabetes in the development of type 2 diabetes in women but not men. This difference between men and women might be attributed to the dominant role of men in Iranian families. According to Iran’s Civil Code, men are considered as the head of the household, the breadwinner of the family, and the guardian of women; they are responsible for providing the necessities of women’s lives such as food, shelter, and health care [25]. It has been shown that in Iranian families, men usually do the shopping for food and decide what dish should be cooked [26]. Also, the sex difference may be due to some major limitations and challenges married women face in Iran; for instance, a husband often forbids his wife from joining a sports team, cycling, and exercising outdoors [27]. It is conceivable that the husband’s inactivity may impose more limitations on their spouse’s physical activity [28]. While speculative, future research needs to evaluate the potential impact of gender roles in the findings reported here. The research agenda should include data about marital quality, sexual relationship, and psychological well-being of couples collected using qualitative or open-ended questionnaire. Also, future research would benefit from the use of other factors such as household income, built environment, access to care, and diet quality.

Our findings can have several kinds of implications: (1) the spousal concordance for type 2 diabetes could alert individuals to the potential risk of developing diabetes, especially among women, and (2) spousal diabetes can provide valuable information for the detection of undiagnosed diabetes and also for diabetes screening programs in order to identify high-risk individuals, and last, but not least, our findings can encourage couples to adopt a healthier lifestyle, not only to benefit themselves, but also to promote and maintain the health of their partners.

### Strengths and limitations

Strengths of our study include a large sample size with a long-term follow-up duration. Our study included all married couples that had at least one child after their marriage; hence, we eliminated the bias of self-reported measures of marital status. However, our study has several limitations that should be acknowledged. First, similar to several other published studies, since the information on marital duration was not available, we could not evaluate the effect of shared lifestyle patterns on the observed associations. Second, we analyzed spousal risk factors measured at baseline; these factors might have changed during the study period, and couples might have separated. Lastly, the study was conducted only among Tehranian couples aged ≥ 20 years with at least one child; and therefore results might not be generalized to all couples or to other parts of the country.

## Conclusion

Our study demonstrated that spouses of men with type 2 diabetes were at increased risk of developing diabetes after adjusting for confounders and their own FPG level. The risk did not drastically diminish when we adjusted for woman’s own BMI, suggesting that among women, the impact of spousal diabetes on the risk of type 2 diabetes might be mediated by other unhealthy behaviors such as physical inactivity and unhealthy eating habits. Our findings indicate the importance of shared environmental factors in the development of type 2 diabetes. Moreover, our results might contribute to the early detection of individuals at high risk of developing type 2 diabetes, particularly, among women.

### Perspectives and significance

The present study provides evidence that having a husband with diabetes increases an individual’s risk of developing diabetes even beyond the effect of the individual’s own risk factors for diabetes. Study findings also suggest the importance of shared environmental factors during the development of diabetes. This relationship requires adopting comprehensive study design and including a large number of lifestyle factors in order to fully understand the concordance mechanism, but it may have implications for the early detection of high-risk individuals or undiagnosed diabetes, especially in the screening and intervention programs.

## Data Availability

Datasets analyzed for the current study are available from the corresponding author on reasonable request.
